# Lipid profiling suggests species specificity and minimal seasonal variation in Pacific Green and Hawksbill Turtle plasma

**DOI:** 10.1371/journal.pone.0253916

**Published:** 2021-07-19

**Authors:** Chelsea E. Clyde-Brockway, Christina R. Ferreira, Elizabeth A. Flaherty, Frank V. Paladino

**Affiliations:** 1 Department of Forestry and Natural Resources, Purdue University, West Lafayette, IN, United States of America; 2 Bindley Bioscience Center, Purdue University, West Lafayette, IN, United States of America; 3 Department of Biology, Purdue University-Fort Wayne, Fort Wayne, IN, United States of America; Ocean Frontier Institute, CANADA

## Abstract

In this study, we applied multiple reaction monitoring (MRM)-profiling to explore the relative ion intensity of lipid classes in plasma samples from sea turtles in order to profile lipids relevant to sea turtle physiology and investigate how dynamic ocean environments affect these profiles. We collected plasma samples from foraging green (*Chelonia mydas*, n = 28) and hawksbill (*Eretmochelys imbricata*, n = 16) turtles live captured in North Pacific Costa Rica in 2017. From these samples, we identified 623 MRMs belonging to 10 lipid classes (sphingomyelin, phosphatidylcholine, free fatty acid, cholesteryl ester, phosphatidylserine, phosphatidylinositol, phosphatidylglycerol, phosphatidylethanolamine, ceramide, and triacylglyceride) and one metabolite group (acyl-carnitine) present in sea turtle plasma. The relative ion intensities of most lipids (80%) were consistent between species, across seasons, and were not correlated to body size or estimated sex. Of the differences we observed, the most pronounced was the differences in relative ion intensity between species. We identified 123 lipids that had species-specific relative ion intensities. While some of this variability is likely due to green and hawksbill turtles consuming different food items, we found indications of a phylogenetic component as well. Of these, we identified 47 lipids that varied by season, most belonging to the structural phospholipid classes. Overall, more lipids (n = 39) had higher relative ion intensity in the upwelling (colder) season compared to the non-upwelling season (n = 8). Further, we found more variability in hawksbill turtles than green turtles. Here, we provide the framework in which to apply future lipid profiling in the assessment of health, physiology, and behavior in endangered sea turtles.

## Introduction

Wildlife face diverse and complicated anthropogenic threats, in addition to naturally occurring environmental stressors. Anthropogenic threats to sea turtles are widely studied and clearly defined [1], but to adequately protect these endangered species, it is important to understand how natural environmental variability influences their behavior and physiology. Animal behavior and physiology are reflected in plasma metabolites, such as lipid profiles, which also contain information about an organism’s diet, environment, and health [2–8]. Generally, lipids can be divided into structural lipids, storage lipids, and signaling lipids. Structural lipids make up cell membranes with chain lengths and saturation determining membrane fluidity [9–11] and include the phospholipids, specifically sphingomyelins, phosphatidylcholines, phosphatidylserines, phosphatidylinositols, phosphatidylglycerols, and phosphatidylethanolamines. Storage lipids are mostly dietary lipids that are consumed and either oxidized as energy or stored for future energy use, primarily free fatty acids and triacylglycerides [12–14]. Signaling lipids trigger specific cell responses and are precursors for hormones or carrier lipids [15].

Because most sea turtles are threatened or endangered, research has emphasized the understanding of life history patterns to better guide conservation strategies. To date, lipid studies in sea turtles have focused on foraging ecology [6] and reproductive biology [16–18]. However, lipid dynamics can also provide information on other aspects of physiology and behavior, such as chemosensory communication [19–21], oxidative stress associated with migration and feeding [22–24], lung structure and function [25–27], and as indication of disease status [28–31] and aging [32]. Comprehensive lipid-profiling is a powerful exploratory tool that highlights latent aspects of animal ecology and health through characterizations of lipid quantities relative to the whole profile. Further, targeted assessment required to quantify lipid concentrations depends on knowledge of specific lipids, fatty acyl chain composition, and saturation. Therefore, our first objective was to screen lipids and metabolites present in sea turtle plasma, using the Multiple Reaction Monitoring (MRM)-profiling method [33–36]. We present lipid profiles in foraging non-reproductive green (*Chelonia mydas*, Linnaeus, 1758) and hawksbill (*Eretmochelys imbricata*, Linnaeus, 1766) sea turtles from Costa Rica, including specific chain lengths and saturation levels during normal El Niño conditions [37]. Subsequently, we quantified how relative ion intensity of lipid profiles varied between species of sea turtles and across seasons to determine if phylogeny, diet, size, or temperature-influenced lipid profiles. Understanding how turtles relate to their dynamic environments will allow for specific conservation efforts better tailored to sea turtle’s needs.

## Methods

### Ethics statement

This study was carried out in accordance with institutional, federal, and international guidelines and permits. All data were collected under the protocol approved by Purdue Animal Care and use Committee (protocol #1510001309). Permissions to work with endangered sea turtles in Costa Rica were granted under permits from Guanacaste Conservation Areas (ACG) of the Ministry of Environment of Costa Rica (ACG-PI-PC-019, R-ACG-057-2016). Finally, samples were approved for importation into the United States by USFWS and CITES (CITES permit 17US06369C/9).

### Study area

We conducted this study during 2017 in Matapalito Bay (10.93°N; -85.79°W) and Salinas Bay (11.04°N; -85.70°W) in North Pacific Costa Rica. This area represents one of three major upwelling areas along the Central American coast [38]. Costa Rican upwelling is especially strong because of the interaction between trade winds and Costa Rica Dome water patterns [38–40]. Therefore, sea turtles inhabiting these waters are exposed to unusually cold-water temperatures (< 20°C at 10 m) during the upwelling season (UP; November–March), and warmer temperatures (~28°C at 10 m) during the non-upwelling season (NUP; April–October). The transition time between seasons is not definite and varies between years. Additionally, the Eastern Pacific is exposed to multiannual (~4 yr) water cycles known as the El Niño Southern Oscillation (ENSO). During El Niño years, water temperatures are warmer than usual (decreased primary productivity) while La Niña years are characterized by colder than normal temperature and increased upwelling [37]. In this study, we collected samples during normal El Niño conditions (https://ggweather.com/enso/oni.htm).

### Study animals

In North Pacific Costa Rica, black and yellow morphotype green turtles (*Chelonia mydas*), collectively referred to as green turtles, and hawksbill turtles (*Eretmochelys imbricata*), inhabit coastal sites concurrently [41]. We live captured black (8 juvenile, 6 male, 3 female), yellow (9 juvenile, 2 female), and hawksbill (15 juvenile, 1 female) turtles using tangle nets [42] deployed in each bay once a month; however, we canceled the monthly sampling during dangerous weather. We opportunistically hand-caught turtles encountered when snorkeling. We brought turtles into the boat for processing, where we measured curved carapace length measured from the nuchal notch to the most posterior tip of the caudal peduncle (CCL; flexible measuring tape; ± 0.5 cm), and mass (Detecto 11S200HKG “S” hook hanging scale, Webb City, MO; ± 0.5 kg). To identify individuals and avoid pseudo-replication, we marked turtles with a unique passive integrated transponder (PIT) tag injected into the right shoulder (AVID2028 FriendChip, Norco, California, USA), and metal flipper tags (Style 681IC, National Band and Tag Company, Newport, KY, USA) in both hind flippers [43]. We grouped individuals into age/sex class by size, where juvenile green turtles had CCL ≤ 76 cm and adult turtles had a CCL > 76 cm, because the smallest turtle in this study with defining male characteristics was 77 cm CCL. This classification combined what other studies classified as sub-adult and adult classes [44, 45]. In hawksbill turtles, we classified juveniles as < 70 cm CCL and adults as > 71 cm CCL [46, 47].

### Plasma collection and preparation

We collected a single blood sample (< 1 ml/kg) per individual using a 21 g non-heparinized needle from the cervical sinus [48], transferred the sample to a lithium-heparin tube, and stored it on ice until returned to the lab. Whole blood samples were centrifuged for 5 min at 1145 g (CAT No. 0151, Clay Adams Analytical Centrifuge, New York, NY). We isolated the plasma in a non-heparinized tube, and stored the plasma at -18°C for up to 1 year until we transported samples back to Purdue University. Samples were then subsequently stored at -20°C until analyses (within ~9 months).

### Lipid analysis

For lipid identification, we used a recently published strategy, MRM-profiling mass spectrometry, which is a two-step process to identify lipid profiles [33–36]. The first step involved generating and analyzing a reference sample, for this step we combined 12.5 μL from 16 hawksbill turtles and 16 green turtles (randomly selected) to generate two 200 μL reference samples. We extracted lipids and fat-soluble metabolites from plasma samples following the chloroform-methanol Bligh and Dyer [49] lipid extraction procedure and stored extracted lipids at -80°C until analysis. For analysis, we resuspended dried samples in ACN+MeOH+300nM NH_4_Ac 3:6.65:0.35 (v/v).

We compiled a set of methods by combining *m/z* for all molecular ions based on the LipidMAPS online database (http://www.lipidmaps.org/) with expected ions resulting from the Prec or NL scans (1,412 lipids). We combined all potential MRMs into 11 methods (no more than 200 MRM per method). For each method, we used a capillary pump connected to the autosampler (G1377A) then connected to an Agilent 6410 QQQ mass spectrometer (Agilent Technologies, Santa Clara, CA, USA). One sample injection was performed to profile each lipid class. We processed the raw MRM mass spectrometry data using an in-house script and MRM transitions and exported the resulting ion intensity values to Microsoft Excel (v2016, Microsoft Corporation, Redmond, WA). We normalized the absolute ion intensity of lipids against the total ion current of the method for each turtle individually. We then selected any lipid that had a sample ion intensity higher than the blank, resulting in 623 baseline lipids and metabolites from 10 lipid classes and one metabolite class and combined these 623 lipids and metabolites into four methods.

This allowed us to identify and process only lipids and metabolites with ion intensities greater than that of the blank, suggesting significance to sea turtle physiology. The second step involved analyzing the individual turtle plasma samples for the identified baseline lipids. We used the same procedure except we modified the extraction procedure to work with 20 μL of plasma (due to sensitivity of the technique). MRM lipid-profiling provides information on diverse lipid classes (sphingomyelin, phosphatidylcholine, free fatty acid, cholesteryl ester, phosphatidylserine, phosphatidylinositol, phosphatidylglycerol, phosphatidylethanolamine, ceramide, and triacylglyceride), one metabolite group (acyl-carnitine), and aids in screening for shifts in lipid physiology that indicate environmental pressures, metabolic impairments, and resource allocation [14, 15, 28, 29, 33]. Even though the method proportionates tentative identification of the lipids at the species level, it allows a comprehensive lipid profiling and the understanding of the most important lipid classes and specific candidates related to a physiological or pathological condition. While we normalized each turtle profile by method in step one, here we normalized the absolute ion intensity of lipids and metabolites against the total ion current of the lipid class for each turtle. The resulting relative ion intensity of lipids, therefore, represented the proportion of total lipid class ion current for each turtle. For the acyl-carnitine metabolite class, we only included the biologically significant lipid fragment (85.1).

### Statistical analysis

We conducted univariate and multivariate analyses on relative ion abundances using MetaboAnalyst 4.0 (http://www.metaboanalyst.ca, Rv3.4.3 [50, 51]). For each analysis, we normalized the data within MetaboAnalyst using the auto-scaling method (mean-centered and divided by the standard deviation of each variable). As an overview, we used principal component analyses and a partial least-squares discriminant analyses to evaluate clustering of turtles based on lipid profiles. Within MetaboAnalyst, we used the permutation test function with 1000 iterations and the cross-validation (CV) results to determine if the partial least-squares discriminant analysis (PLS-DA) model fit [52]. If our partial least-squares discriminant analysis permutations test was not significant or CV lacked predictive ability, we used our principal component analysis model. To identify differential lipids, we used Fold Change analysis (FC >2; principal component analyses data) or variable importance in the projection (VIP; partial least-squares discriminant analyses data) and univariate analyses (two-group data: t-test and fold change analysis; multi-group: one-way ANOVA with Fisher’s Least Significant Difference post hoc analysis), for each lipid or metabolite class separately. We classified differential lipids as those with VIP values > 1 or fold change threshold of 2, and significant univariate test results (p < 0.05). To investigate the effects of turtle size (curved carapace length and body mass), we conduced Pearson R correlations. We classified lipids into 5 categories based on correlation coefficient (r): strong negative correlation (-1.0 –-0.6), weak negative correlation (-0.59 –-0.4), no correlation (-0.39–0.39), weak positive correlation (0.4–0.59), and strong positive correlation (0.6–1.0).

We analyzed each lipid/metabolite class separately. First, we compared black turtles between bays and determined no significant difference across habitats. Therefore, for subsequent analyses we combined turtles from both bays. Next, we tested seasonal effects in black turtles (upwelling: November–March; non-upwelling: April–October). If we did not identify differential lipids between seasons, then we combined all black turtles and compared relative ion intensity for each lipid class between black and yellow morphotype green turtles and all green turtles across seasons. Assuming statistical similarity within all green turtles, we compared green turtles to hawksbill turtles and all individuals across seasons. When groups were not statistically similar, we used a one-way ANOVA to compare species and seasons simultaneously by dividing the data into 4 or 6 groups, depending on the analysis (by population and season). Further, we compared turtle size (curved carapace length and mass) between populations and across seasons and locations using Two-Way Analysis of Variance (ANOVA; black turtles), One-Way Analysis of Variance (ANOVA; yellow turtles) or Kruskal-Wallis tests (hawksbill turtles, population) in R statistical software (Version 3.4.4, [53]) and tested sex/age class differences in lipid relative ion intensity. In all analyses, we considered statistical significance as a threshold of p < 0.05. Because we were using relative ion intensity, higher values suggested more lipid present in plasma, however this was not comparable to concentrations. Further, we built our profiles by lipid class, therefore relative ion intensities could not be compared across lipid classes, only within classes.

Besides evaluating individual (univariate statistics) and a combination of lipids (multivariate statistics), the diversity of lipid structures present in the sea turtle plasma was relative to total fatty acyl chain length and unsaturation levels. For that, we divided each lipid class by carbon chain length (short, medium, long) and saturation level (0 = unsaturated, 1–2 = mono and di-unsaturated lipids, 3+ = polyunsaturated lipids) and presented the results as percent (%) of lipid class profile.

## Results

We analyzed 44 plasma samples from sea turtles ranging from 38.5–92.0 cm curved carapace length and 6.0–88.0 kg body mass (Table 1). Due to scale malfunction, we recorded body mass of 39 turtles (out of 44 turtles; Table 1). Throughout 2017, we captured 26 turtles during the non-upwelling season and 19 turtles during the upwelling season (Table 1). Turtle length and body mass did not vary with season (F_1,14_ = 0.706, p = 0.415 and F_1,12_ = 0.158, p = 0.698, respectively) or location (F_1,14_ = 0.007, p = 0.934 and F_1,12_ = 0.000, p = 0.996, respectively) in black morphotype green sea turtles, or by season in yellow morphotype (F_1,9_ = 0.483, p = 0.505 and F_1,5_ = 0, p = 0.998, respectively) and hawksbill turtles (χ^2^_1_ = 1.697, p = 0.193 and χ^2^_1_ = 1.381, p = 0.24, respectively). However, the three populations were significantly different in curved carapace length (χ^2^_2_ = 24.694, p < 0.001) and body mass (χ^2^_2_ = 23.022, p < 0.001).

**Table 1:** Morphological measurements and capture data of sea turtles foraging in Pacific Costa Rica.

	CCL (cm)	CCL range	Mass (kg)*	Mass range*	n	NUP Season	UP Season
Black	77.5 ± 7.0	66.0–92.0	49.5 ± 14.5	32.0–88.0	17	11	6
Yellow	66.5 ± 10.0	50.0–80.5	30.5 ± 17.5	13.0–66.0	11	3	8
Hawksbill	51.0 ± 11.5	38.5–83.0	13.0 ± 11.5	6.0–50.0	16	12	5

Morphological measurements of sea turtles sampled including curved carapace length (CCL) in cm, and body mass in kg, with values representing mean ± standard deviation and ranges. Non-upwelling (NUP) season (April–October) represent the number of turtles captured during the non-upwelling season, while upwelling (UP) season (November–March) represent the number of turtles captured during the upwelling season. Black = black morphotype green turtles, Yellow = yellow morphotype green turtles.

*Mass measurements presented for Black n = 16, Yellow n = 7, and Hawksbill n = 16.

### Lipid and metabolite profiles

**Fig 1:**
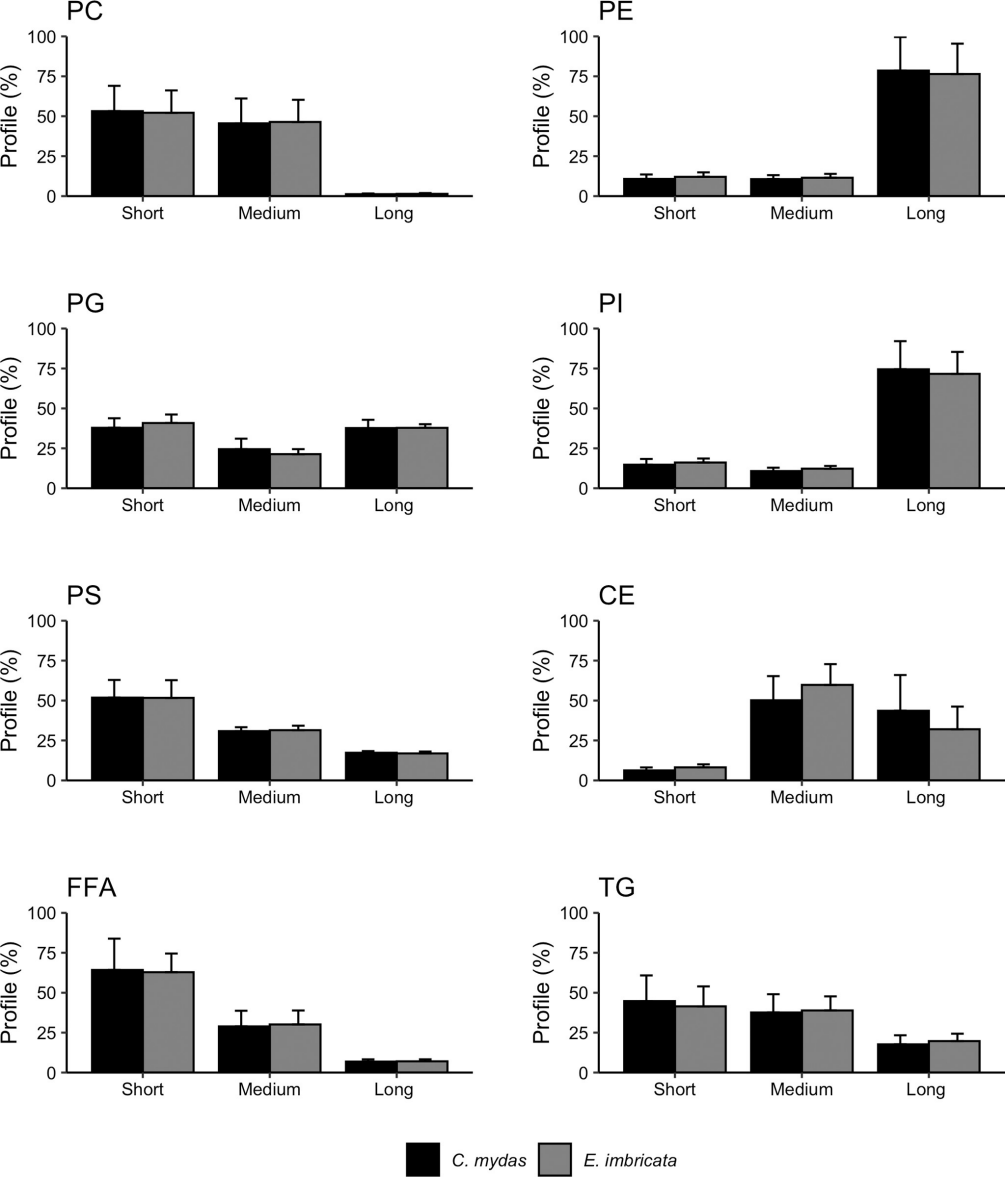
Percent lipid class profile from sea turtles divided by chain length and species. The x-axis represents chain length and was divided based on each profile separately. Bars and error bars represent mean ± standard deviation (%). We did not include sphingomyelin, acyl-carnitine, or ceramide classes due to structure. PC = phosphatidylserine (short = 30–36 carbons, medium = 38–42 carbons, long = 44–48 carbons); PE = phosphatidylethanolamine (short = 12–22 carbons, medium = 24–34 carbons, long = 36–42 carbons); PG = phosphatidylglycerol (short = 12–22 carbons, medium = 24–34 carbons, long = 36–44 carbons); PI = phosphatidylinositol (short = 12–22 carbons, medium = 24–34 carbons, long = 36–44 carbons); PS = phosphatidylserine (short = 14–24 carbons, medium = 26–36 carbons, long = 38–44 carbons); CE = cholesteryl ester (short = 12–15 carbons, medium = 16–19 carbons, long = 20–24 carbons); FFA = free fatty acid (short = 12–18 carbons, medium = 20–26 carbons, long = 28–34 carbons); TG = triacylglyceride (short = 48–50 carbons, medium = 52–54 carbons, long = 56–60 carbons).

**Fig 2:**
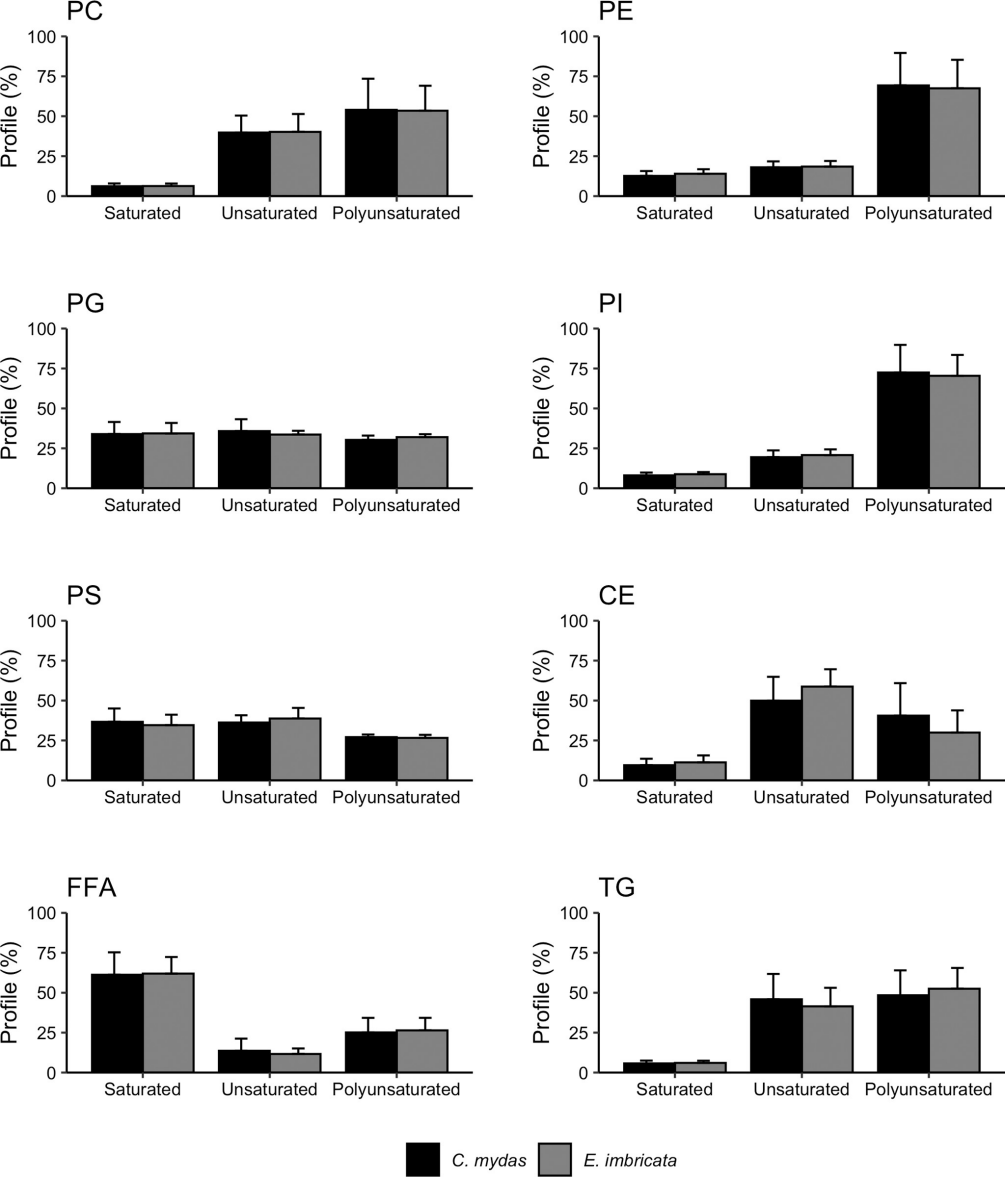
Percent lipid class profile from sea turtles divided by saturation and species. Bars and error bars represent mean ± standard deviation (%). The x-axis represents saturation level where saturated indicates lipids 0 double bonds, unsaturated indicates lipids with 1 or 2 double bonds, and polyunsaturated indicates lipids with ≥3 double bonds. We did not include sphingomyelin, acyl-carnitine, or ceramide classes due to structure.

We identified 623 baseline MRMs (referred as lipids or acyl-carnitines; all attributions are tentative) from 11 classes by using their relative ion intensities. These lipids and acyl-carnitines presented ion intensities higher than the blank sample in the plasma samples of the sea turtles (Table 2 and S1 Table). Among the structural lipids, most of the phosphatidylcholine and phosphatidylserine class profile consisted of short chain lipids (> 50%) while most of the phosphatidylethanolamine and phosphatidylinositol class profile consisted of long chain lipids (> 75%; Fig 1). The phosphatidylglycerol profile was evenly distributed across lipid lengths. Among the dietary, signaling, and fat storage lipids, short chain lipids comprised a higher percentage of total lipid profile in free fatty acids and triglycerides while most cholesteryl ester profile was comprised of medium to long lipid lengths (Fig 1). Saturation graphs revealed a higher percentage of unsaturated lipids in our phosphatidylcholine, phosphatidylethanolamine, and phosphatidylinositol profiles with no difference in saturation across phosphatidylglycerol and phosphatidylserine profiles (Fig 2). In our dietary and storage lipids, most cholesteryl ester and triglyceride lipids were unsaturated while most free fatty acids were saturated (Fig 2). We did not include sphingomyelin, acyl-carnitine, or ceramide classes due to structure (S1 Table).

**Table 2:** Plasma lipid and metabolite classes isolated from sea turtle plasma, and correlation to length and mass.

Class	Baseline lipids	Differential lipids	By Species	By Season	Correlation Coefficient CCL				Correlation Coefficient Body Mass	
					Strong -	Weak -	Weak +	Strong +	Weak -	Weak +
PC	113	26	22	17	-	7	6	-	1	3
SM	27	14	10	12	-	3	-	2	-	1
CE	55	20	20	2	-	5	2	-	-	3
TG	165	44	44	6	4	27	13	-	2	6
PI	50	9	9	5	-	7	1	-	4	-
PE	55	3	3	2	-	2	1	-	-	-
Cer	27	4	4	2	-	1	-	-	1	1
FFA	26	2	2	1	-	1	1	-	-	-
PG	33	1	1	-	-	7	1	-	-	-
PS	24	-	-	-	-	-	-	-	1	1
Car	48	-	-	-	-	-	-	-	-	-

Sea turtle plasma lipid and metabolite classes and their correlation to curved carapace length (CCL, cm) and body mass (kg). Baseline lipids are those lipids and metabolites with relative ion intensities greater than the blank. Differential lipids are those lipids and metabolites identified by comparative analyses between species and season. Columns labeled “by species” and “by season” indicate the number of lipids that had significantly different relative ion intensities, (-) indicates no data or no correlation. All turtles were live captured in Costa Rica in 2017. Sphingomyelin (SM), Phosphatidylcholine (PC), Free Fatty Acids (FFA), Cholesteryl Esters (CE), Acyl-carnitines (Car), Phosphatidylserine (PS), Phosphatidylinositol (PI), Phosphatidylglycerol (PG), Phosphatidylethanolamine (PE), Ceramide (Cer), and Triacylglyceride (TG).

**Fig 3:**
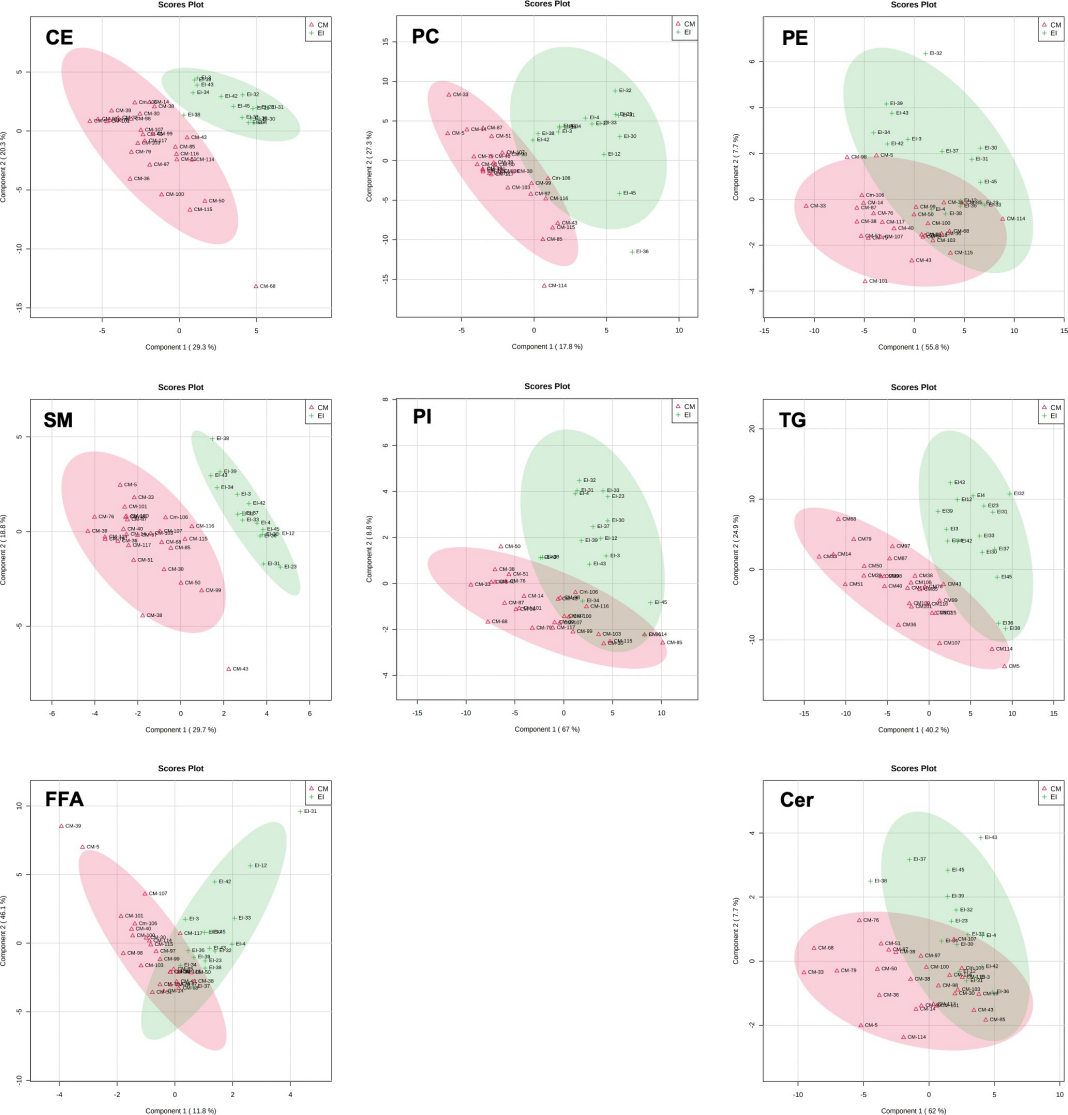
Partial least-squares discriminant analysis of species clusters by plasma lipid profiles. Partial least-squares discriminant analysis (PLS-DA) component 1 (x-axis) and component 2 (y-axis) demonstrating species clusters as a result of plasma lipid profile in green turtles (CM, red triangle) and hawksbill turtles (EI, green plus sign). We live captured all turtles in North Pacific Costa Rica in 2017. Abbreviations: CE = cholesteryl ester; PC = phosphatidylcholine; PE = phosphatidylethanolamine; SM = sphingomyelin; PI = phosphatidylinositol; TG = triacylglyceride; Cer = ceramide; FFA = free fatty acids; PG = phosphatidylglycerol; PS = phosphatidylserine; Car = acyl-carnitine.

**Fig 4:**
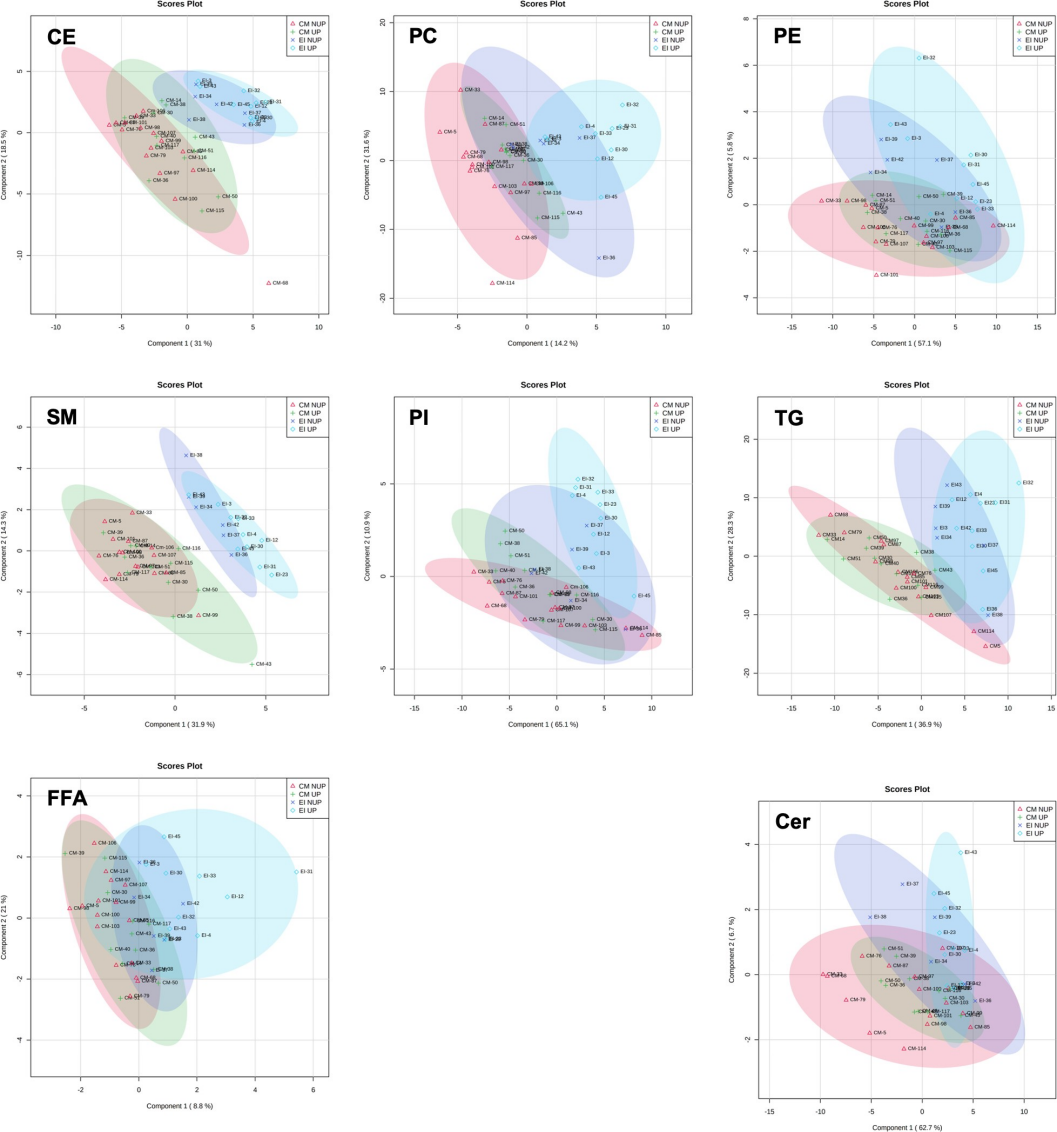
Partial least-squares discriminant analysis displaying seasonal clusters by plasma lipid profiles. Partial least-squares discriminant analysis (PLS-DA) component 1 (x-axis) and component 2 (y-axis) demonstrating species clusters as a result of plasma lipid profile in green turtles (CM) and hawksbill turtles (EI) and season (NUP = non-upwelling; UP = upwelling). We live captured all turtles in North Pacific Costa Rica in 2017. Abbreviations: CE = cholesteryl ester; PC = phosphatidylcholine; PE = phosphatidylethanolamine; SM = sphingomyelin; PI = phosphatidylinositol; TG = triacylglyceride; Cer = ceramide; FFA = free fatty acids; PG = phosphatidylglycerol; PS = phosphatidylserine; Car = acyl-carnitine.

Most (500) baseline lipids had no relationship with turtle size. Of the 115 lipids that had significant correlation coefficients (r), we identified 64 that were negatively correlated to curved carapace length and 9 lipids that were negatively correlated to body mass (Table 2). Additionally, we identified 27 lipids that were positively correlated to curved carapace length and 15 that were positively correlated to body mass; 14 lipids were correlated to both curved carapace length and body mass (Table 2). We did not identify any correlation between acyl-carnitines (a metabolite class) and curved carapace length or body mass (Table 2). Results from our partial least-squares discriminant analyses revealed clear differentiation between green turtles and hawksbill turtles (Fig 3). When we included season (NUP = non-upwelling; UP = upwelling), we found slight deviation from clustering based on species (Fig 4). In the phosphatidylcholine class, which had 8 lipids that varied between seasons in hawksbills and 5 lipids that varies by season in green turtles (Table 4), both PLS-DA scores plots showed that the non-upwelling season had a larger cluster shift to the left. In the sphingomyelin class, which had 8 lipids that varied by season in hawksbill turtles and 5 lipids that varies by season in green turtles, the upwelling season had a larger spread in green turtles and similar cluster size but shift to right in hawksbill turtles (Fig 4).

### Differential lipids

We defined differential lipids as those that presented significantly different relative ion intensities between species or season (Table 2). Our univariate analyses and VIP/Fold-change scores identified 123 differential lipids (MRMs): 115 that demonstrated species specificity and 47 that varied by seasons (some lipids varied by species and season; Table 2). Habitat was not significant for any lipid or metabolite, therefore black turtles from both sites were combined in our analyses. Further, we did not identify any sex/age class differences in relative ion intensity. Species variability was lipid specific and hawksbill turtles had higher relative ion intensities in more lipids (Table 3). Additionally, we discovered that relative ion intensities of differential lipids were higher in the upwelling season compared to the non-upwelling season (Table 4 and S2 Table). While most lipids did not vary within green turtle morphotypes, 19 lipids in the phosphatidylcholine class varied between seasons in black morphotype green turtles or between black and yellow morphotype green turtles (S2 Table). One lipid in the phosphatidylglycerol class (PG 32:0) had a higher relative ion intensity in black morphotype green turtles compared to yellow morphotype green turtles. Finally, five sphingomyelin lipids had higher average relative ion intensities in the upwelling season in black morphotype green turtles (S2 Table). The acyl-carnitine and phosphatidylserine classes displayed no species or seasonal variability in relative ion intensity.

**Table 3:** Plasma lipids that varied between green and hawksbill turtles.

Tentative Lipid ID	Green Turtle	Hawksbill Turtle	Test	p
PC (34:2)	0.013 ± 0.007	0.025 ± 0.007	F = 15.123	< 0.001
PCo(34:1)	0.020 ± 0.009	0.045 ± 0.034	F = 4.84	0.002
PCo(34:0)	0.003 ± 0.001	0.005 ± 0.003	F = 3.87	0.006
PCo(36:5)	0.012 ± 0.005	0.007 ± 0.003	F = 4.072	0.005
PC (36:5)	0.024 ± 0.016	0.014 ± 0.005	F = 10.212	< 0.001
PC (36:1)	0.045 ± 0.012	0.030 ± 0.006	F = 8.457	< 0.001
PCo(38:6)	0.017 ± 0.006	0.024 ± 0.007	F = 3.788	0.007
PCo(38:5)	0.028 ± 0.010	0.017 ± 0.007	F = 6.425	< 0.001
PC (38:7)	0.002 ± 0.001	0.004 ± 0.001	F = 9.94	< 0.001
PCo(38:0)	0.002 ± 0.001	0.004 ± 0.002	F = 9.427	< 0.001
PC (38:6)	0.024 ± 0.011	0.050 ± 0.015	F = 13.145	< 0.001
PC (38:5)	0.054 ± 0.026	0.038 ± 0.013	F = 8.147	< 0.001
SM (d18:1/12:0)	0.099 ± 0.023	0.018 ± 0.004	t = 18.887	< 0.001
SM (d18:0/12:0)	0.009 ± 0.002	0.003 ± 0.000	F = 111.36	< 0.001
SM (d18:0/18:0)	0.006 ± 0.002	0.008 ± 0.001	t = -5.354	< 0.001
SM (d16:1/22:1)	0.004 ± 0.002	0.006 ± 0.003	F = 6.498	0.001
SM (d18:1/20:0)	0.014 ± 0.006	0.025 ± 0.008	F = 17.118	< 0.001
SM (d18:0/20:0)	0.085 ± 0.019	0.112 ± 0.030	F = 11.327	< 0.001
SM (d18:1/24:1)	0.100 ± 0.019	0.130 ± 0.017	F = 12.051	< 0.001
SM (d18:1/24:0)	0.022 ± 0.005	0.029 ± 0.004	t = -4.73	< 0.001
SM (d18:0/24:0)	0.004 ± 0.001	0.005 ± 0.001	F = 7.119	0.001
SM (d18:1/26:0)	0.002 ± 0.001	0.003 ± 0.001	F = 5.846	0.002
12:0 Cholesteryl ester	0.004 ± 0.001	0.003 ± 0.000	t = 3.699	< 0.001
14:1 Cholesteryl ester	0.003 ± 0.001	0.004 ± 0.001	t = -4.299	< 0.001
16:2 Cholesteryl ester, zymosteryl palmitoleate	0.004 ± 0.001	0.004 ± 0.001	F = 7.254	< 0.001
16:1 Cholesteryl ester	0.029 ± 0.008	0.043 ± 0.007	t = -6.023	< 0.001
15:1 Campesteryl ester	0.030 ± 0.008	0.046 ± 0.007	t = -6.544	< 0.001
16:0 Cholesteryl ester	0.014 ± 0.006	0.018 ± 0.007	t = -2.808	0.008
17:1 Cholesteryl ester; 16:1 Campesteryl ester	0.015 ± 0.007	0.022 ± 0.006	t = -3.369	0.002
17:0 Cholesteryl ester	0.002 ± 0.001	0.004 ± 0.002	t = -2.718	0.010
18:3 Cholesteryl ester, 16:2 Stigmasteryl ester, 16:3 Sitosteryl ester	0.019 ± 0.013	0.032 ± 0.016	F = 4.759	0.006
18:2 Cholesteryl ester, zymosteryl oleate, 16:1 Stigmasteryl ester, 16:2 Sitosteryl ester	0.036 ± 0.011	0.070 ± 0.028	t = -5.763	< 0.001
lanosteryl palmitoleate, 18:2 Campesteryl ester	0.003 ± 0.002	0.005 ± 0.002	t = -3.125	0.003
20:5 Cholesteryl ester	0.129 ± 0.074	0.039 ± 0.017	t = 4.74	< 0.001
20:4 Cholesteryl ester, 18:3 Stigmasteryl ester	0.137 ± 0.046	0.073 ± 0.030	t = 4.997	< 0.001
20:2 Cholesteryl ester, 18:1 Stigmasteryl ester, 18:2 Sitosteryl ester	0.004 ± 0.002	0.005 ± 0.001	t = -2.644	0.011
Cholesteryl nitrolinoleate	0.003 ± 0.001	0.004 ± 0.001	t = -3.357	0.002
20:0 Campesteryl ester	0.003 ± 0.002	0.004 ± 0.001	t = -2.481	0.017
22:6 Cholesteryl ester	0.048 ± 0.030	0.077 ± 0.039	t = -2.743	0.009
22:4 Cholesteryl ester, 20:3 Stigmasteryl ester	0.005 ± 0.001	0.009 ± 0.003	t = -4.991	< 0.001
22:1 Cholesteryl ester, 20:0 Stigmasteryl ester, 20:1 Sitosteryl ester	0.003 ± 0.001	0.004 ± 0.001	t = -2.922	0.006
22:3 Stigmasteryl ester	0.004 ± 0.003	0.006 ± 0.003	t = -2.587	0.013
TG 18:1_30:2	0.008 ± 0.002	0.004 ± 0.001	t = 6.6897	< 0.001
TG 18:2_30:1	0.008 ± 0.002	0.004 ± 0.001	t = 6.5387	< 0.001
TG 16:1_32:2	0.004 ± 0.001	0.004 ± 0.002	t = -4.0824	< 0.001
TG 18:0_30:2	0.005 ± 0.001	0.003 ± 0.001	t = 6.3271	< 0.001
TG 18:1_30:1	0.089 ± 0.042	0.010 ± 0.003	t = 7.3508	< 0.001
TG 16:0_32:2	0.006 ± 0.001	0.011 ± 0.003	t = -8.5017	< 0.001
TG 16:1_32:1	0.008 ± 0.002	0.011 ± 0.003	t = -3.5662	0.001
TG 18:0_30:1	0.003 ± 0.015	0.005 ± 0.001	t = 6.4761	< 0.001
TG 18:1_30:0	0.037 ± 0.014	0.019 ± 0.008	t = 4.8097	< 0.001
TG 16:0_32:1	0.028 ± 0.008	0.043 ± 0.014	t = -4.5956	< 0.001
TG 16:1_32:0	0.006 ± 0.001	0.009 ± 0.002	t = -6.9262	< 0.001
TG 18:0_30:0	0.009 ± 0.002	0.006 ± 0.001	t = 5.0595	< 0.001
TG 16:1_34:3	0.005 ± 0.001	0.008 ± 0.002	t = -7.4713	< 0.001
TG 18:1_32:2	0.037 ± 0.014	0.023 ± 0.010	t = 3.4106	0.001
TG 18:2_32:1	0.005 ± 0.002	0.010 ± 0.004	t = -4.8916	< 0.001
TG 16:0_34:3	0.014 ± 0.005	0.031 ± 0.012	t = -6.4608	< 0.001
TG 16:1_34:2	0.008 ± 0.002	0.016 ± 0.005	t = -7.2389	< 0.001
TG 18:1_32:1	0.022 ± 0.005	0.028 ± 0.006	t = -3.4009	0.001
TG 16:0_34:2	0.034 ± 0.013	0.076 ± 0.025	t = -7.4536	< 0.001
TG 16:1_34:1	0.004 ± 0.001	0.006 ± 0.002	t = -6.2444	< 0.001
TG 18:1_32:0	0.004 ± 0.001	0.005 ± 0.001	t = -4.1461	< 0.001
TG 16:0_34:0	0.008 ± 0.002	0.011 ± 0.002	t = -4.7321	< 0.001
TG 18:1_34:5	0.005 ± 0.002	0.003 ± 0.001	t = 4.1061	< 0.001
TG 16:1_36:4	0.003 ± 0.001	0.004 ± 0.001	t = -2.7554	0.009
TG 16:1_36:3	0.003 ± 0.001	0.004 ± 0.001	t = -3.8935	< 0.001
TG 16:0_36:3	0.006 ± 0.002	0.009 ± 0.002	t = -2.778	0.008
TG 18:0_34:2	0.005 ± 0.001	0.006 ± 0.001	t = -3.7369	0.001
TG 16:1_36:1	0.005 ± 0.001	0.006 ± 0.001	t = -2.6626	0.011
TG 18:0_34:1	0.009 ± 0.001	0.011 ± 0.003	t = -2.8509	0.007
TG 16:0_36:1	0.011 ± 0.001	0.013 ± 0.003	t = -2.6241	0.012
TG 16:1_38:6	0.003 ± 0.001	0.004 ± 0.001	t = -3.3506	0.002
TG 16:1_38:5	0.003 ± 0.001	0.004 ± 0.001	t = -3.5319	0.001
TG 16:1_38:4	0.003 ± 0.001	0.004 ± 0.001	t = -3.7781	< 0.001
TG 20:4_34:0	0.005 ± 0.001	0.006 ± 0.001	t = -2.8705	0.006
TG 18:0_36:4	0.003 ± 0.001	0.004 ± 0.001	t = -2.5985	0.013
TG 16:0_38:4	0.004 ± 0.001	0.006 ± 0.001	t = -4.6742	< 0.001
TG 16:0_38:3	0.003 ± 0.001	0.004 ± 0.001	t = -2.9086	0.006
TG 16:0_38:2	0.003 ± 0.001	0.004 ± 0.001	t = -2.7028	0.01
TG 16:0_40:7	0.003 ± 0.001	0.004 ± 0.001	t = -2.8966	0.006
TG 16:0_40:6	0.003 ± 0.001	0.004 ± 0.001	t = -3.0631	0.004
TG 18:1_38:4	0.003 ± 0.001	0.004 ± 0.001	t = -3.5702	0.001
TG 16:0_40:5	0.003 ± 0.001	0.004 ± 0.001	t = -2.882	0.006
TG 18:0_38:4	0.003 ± 0.001	0.004 ± 0.001	t = -2.8917	0.006
PI (34:2)	0.014 ± 0.002	0.016 ± 0.002	F = 7.77	< 0.001
PI (34:1)	0.017 ± 0.003	0.021 ± 0.004	F = 5.055	0.005
PI (36:4)	0.023 ± 0.005	0.030 ± 0.007	F = 10.64	< 0.001
PI (36:3)	0.018 ± 0.004	0.022 ± 0.004	F = 6.402	0.001
PI (38:6)	0.014 ± 0.003	0.017 ± 0.003	F = 6.727	0.001
PI (38:5)	0.073 ± 0.031	0.043 ± 0.011	F = 7.739	< 0.001
PI (38:2)	0.012 ± 0.002	0.014 ± 0.001	t = -2.874	0.006
PI (40:6)	0.016 ± 0.003	0.021 ± 0.003	t = -5.137	< 0.001
PI (40:4)	0.015 ± 0.002	0.021 ± 0.004	t = -7.054	< 0.001
PEp (36:6)	0.014 ± 0.002	0.016 ± 0.003	t = -3.409	0.001
PE (36:0)	0.014 ± 0.002	0.017 ± 0.003	t = -3.868	< 0.001
PE (38:5)	0.578 ± 0.028	0.035 ± 0.011	t = 3.263	0.002
Cer(d18:2/16:0)	0.031 ± 0.003	0.032 ± 0.002	F = 4.66	0.007
CerP(d18:1/16:0)	0.030 ± 0.003	0.034 ± 0.004	t = -3.453	0.001
Cer(d18:1/24:1(15Z))	0.107 ± 0.032	0.080 ± 0.026	F = 4.684	0.007
CerP(d18:1/24:1(15Z))	0.030 ± 0.003	0.033 ± 0.004	F = 5.65	0.003
FFA C22:6; C22:6	0.034 ± 0.005	0.048 ± 0.021	F = 7.282	0.001
FFA C22:4; C22:4	0.023 ± 0.003	0.032 ± 0.010	t = -4.249	< 0.001

Average relative ion intensity of differential plasma lipids that varied by species (green turtles and hawksbill turtles) live captured in Costa Rica in 2017. Not shown here is that 15 lipids varied by morphotype in green turtles (S2 Table). PC = phosphatidylcholine; SM = sphingomyelin; TG = triacylglyceride*; PI = phosphatidylinositol; PE = phosphatidylethanolamine, Cer = ceramide; FFA = free fatty acids (C = carbon).

*For the profiling of the TG lipids, we have used MRMs containing the expected ammoniated adduct and as the product ion the neutral loss expected for a given fatty acyl chain. This method was inspired by [54]. Since this approach has no class specificity, it may be that other glycerolipids overlap even though they are do not occur in large abundances at the mass range profiled. The nomenclature formatting (e.g TG 18:1_30:2) indicates the class abbreviature (TG) followed by the carbon:unsaturation number for the fatty acid targeted by the neutral loss used to determine the product ion of the MRM (18:1) followed by an underline and the sum of the carbon:unsaturation on the other two fatty acids present in the TG (_30:2).

**Table 4:** Plasma lipids that varied between the upwelling and non-upwelling seasons.

Tentative Lipid ID	Hawksbill NUP	Hawksbill UP	Test	p	Green Turtle NUP	Green Turtle UP	Test	p
PC (30:2)					0.002 ± 0.000	0.002 ± 0.000	F = 7.395	< 0.001
PC (30:1)					0.034 ± 0.010	0.023 ± 0.008	t = -3.527	0.003
PCo(34:1)	0.028 ± 0.024	0.055 ± 0.036	F = 4.84	0.002				
PC (34:2)	0.020 ± 0.008	0.028 ± 0.005	F = 15.123	< 0.001				
PC (38:8)	0.006 ± 0.001	0.004 ± 0.001	F = 7.441	< 0.001	0.004 ± 0.001	0.003 ± 0.001		
PCo(38:1)	0.008 ± 0.002	0.005 ± 0.001	F = 7.125	< 0.001	0.005 ± 0.002	0.004 ± 0.001		
PC (38:7)	0.003 ± 0.001	0.004 ± 0.002	F = 9.94	< 0.001				
PCo(38:0)	0.003 ± 0.001	0.005 ± 0.002	F = 9.427	< 0.001				
PC (38:6)	0.039 ± 0.015	0.057 ± 0.012	F = 13.145	< 0.001				
PC (40:5)	0.016 ± 0.009	0.009 ± 0.003	F = 6.043	< 0.001	0.013 ± 0.006	0.017 ± 0.006		
SM (d18:0/12:0)					0.009 ± 0.001	0.010 ± 0.002	F = 111.36	< 0.001
SM (d18:2/20:1)	0.002 ± 0.001	0.003 ± 0.001	F = 8.276	< 0.001	0.002 ± 0.000	0.002 ± 0.001	F = 8.276	< 0.001
SM (d18:2/22:1)	0.036 ± 0.010	0.048 ± 0.008	F = 6.687	0.001	0.029 ± 0.011	0.042 ± 0.013	F = 6.687	0.001
SM (d18:1/20:0)	0.019 ± 0.007	0.028 ± 0.005	F = 17.118	< 0.001				
SM (d18:1/24:1)	0.142 ± 0.021	0.122 ± 0.007	F = 12.051	< 0.001				
SM (d18:1/26:0)	0.003 ± 0.001	0.003 ± 0.001	F = 5.846	0.002				
SM (d18:0/20:0)	0.087 ± 0.026	0.127 ± 0.021	F = 11.327	< 0.001				
SM (d18:0/24:0)					0.004 ± 0.001	0.005 ± 0.001	F = 7.119	0.001
SM (d18:0/26:0)	0.002 ± 0.001	0.002 ± 0.000	F = 4.278	0.01	0.001 ± 0.000	0.002 ± 0.001	F = 4.278	0.01
SM (d16:1/22:1)	0.003 ± 0.002	0.007 ± 0.002	F = 6.498	0.001				
18:3 Cholesteryl ester, 16:2 Stigmasteryl ester, 16:3 Sitosteryl ester	0.023 ± 0.015	0.037 ± 0.014	F = 4.759	0.006				
16:2 Cholesteryl ester, zymosteryl palmitoleate					0.003 ± 0.001	0.004 ± 0.001	F = 7.254	< 0.001
TG 16:0_32:2	0.010 ± 0.003	0.012 ± 0.002	F = 28.321	< 0.001				
TG 18:0_30:1					0.025 ± 0.013	0.036 ± 0.016	F = 17.539	< 0.001
TG 16:1_32:0	0.007 ± 0.001	0.010 ± 0.002	F = 24.723	< 0.001				
TG 16:1_34:1	0.006 ± 0.001	0.007 ± 0.002	F = 22.687	< 0.001				
TG 16:0_38:4	0.005 ± 0.001	0.007 ± 0.002	F = 9.7085	< 0.001				
PI (34:2)	0.015 ± 0.002	0.017 ± 0.002	F = 7.77	< 0.001				
PI (36:3)	0.019 ± 0.004	0.024 ± 0.003	F = 6.402	0.001				
PI (36:4)								
PI (38:6)	0.014 ± 0.002	0.018 ± 0.002	F = 6.727	0.001				
PI (38:5)					0.062 ± 0.029	0.087 ± 0.028	F = 7.739	< 0.001
PEp (36:6)	0.013 ± 0.003	0.014 ± 0.002	8.318	< 0.001				
PE (36:0)	0.017 ± 0.004	0.016 ± 0.001	7.516	< 0.001				
CerP(d18:1/24:1(15Z))	0.031 ± 0.002	0.034 ± 0.004	F = 5.65	0.003				
Cer(d18:2/16:0)					0.030 ± 0.003	0.032 ± 0.002	F = 4.66	0.007
FFA C22:6; C22:6	0.036 ± 0.004	0.055 ± 0.025	F = 7.282	0.001				

Average relative ion intensity of differential plasma lipids that varied by season (NUP = non-upwelling, and UP = upwelling) in green turtles and hawksbill turtles live captured in Costa Rica in 2017. Some lipids varied only in black morphotype green turtles, S2 Table. PI (36:4) was significantly higher in UP compared to NUP across both species (F = 10.64; p < 0.001). In addition, 16 lipids varied by season in black morphotype green turtles (S2 Table). PC = phosphatidylcholine; SM = sphingomyelin; TG = triacylglyceride*; PI = phosphatidylinositol; PG = phosphatidylglycerol; PE = phosphatidylethanolamine, Cer = ceramide; FFA = free fatty acids (C = carbon).

## Discussion

Establishing metabolomic approaches to studying endangered species is a key step to assess adaptations and facilitate understanding of their physiology in dynamic environments [8, 31, 55, 56]. This has important ecological implications, particularly with changes in ocean temperature and nutrient variability that accompanies global climate change [57, 58]. Using a single plasma sample of 20μL and the MRM-profiling method, we were able to profile major lipid and metabolite classes that are relevant to sea turtle biology. Quantification of lipid and metabolite concentrations in plasma usually requires the use of larger amounts of samples and deuterated standards that are only effective if specific targeted lipids, chain lengths, and saturation levels are known. Therefore, using the data we present here (S1 Table), and data from Ahmadireskety et al. [8] researchers can compare and further investigate concentrations and patterns of lipid and metabolite expression within sea turtles. Further, due to the similarity between lipid species in green and hawksbill turtles (this study) and those in other studies of green, loggerhead (*Caretta caretta*), and leatherback sea turtles (*Dermochelys coriacea*), we predict that our data are applicable to other sea turtle species foraging in other environments throughout world [8, 59].

In our study, fewer than 20% of the lipids identified reflected species differences and/or seasonality in relative ion intensity in sea turtles foraging in Pacific Costa Rica. Among these, the most remarkable difference we observed in relative ion intensities of lipids was between species, suggesting variability is due to diet, genetics/phylogeny or age/sex, and proximity to initiation of migration (Table 3, Fig 3). In tuatara (*Sphenodon punctatus* [60, 61]), lizards (*Amphibolurus nuchalis* [62]), alligators (*Alligator mississippiensis* [63, 64]), frogs (*Leptodactylus fallax* [65]), fish [66], and birds [3, 5, 67] lipid compositions of diets influenced plasma lipid profiles. When foraging within the same habitat, green and hawksbill turtles have minimal overlap in diets [46, 68, 69], therefore diet could explain some of the variability in lipid profiles and partial least-squares discriminant analysis clusters. However, diet would not explain consistency in lipid relative ion intensity found between black and yellow morphotype green turtles. Studies suggest that black and yellow morphotype turtles tend to have distinct diets [46, 70], yet, our partial least-squares discriminant analysis and univariate analyses grouped them together.

Alternatively, in birds (*Anatidae*), phylogeny could be more important than dietary differences (yolk lipids) [71, 72]. While in fish (*L*. *fabricii* and *L*. *maculatus*), total lipid levels are genetically determined while habitat may determine lipid lengths and saturation levels [73]. Similarly, a study on great tits (*P*. *major*) suggests there is an interaction between diet and temperature effects on plasma lipid saturation level, and this effect is reflected in metabolic rate [2]. Therefore, lipid clustering observed in this study likely has a genetic or phylogenetic component similar to nesting green, loggerhead and leatherback turtles in Florida [8], but there may be an interaction effect we did not measure.

We identified size differences between species of turtle in this study; however, our correlation analyses suggest consistency of lipid profiles through age classes with smaller turtles potentially growing at a slightly faster rate than larger turtles (Table 2 [74, 75]). In loggerhead sea turtles (*Caretta caretta*), size related changes in cholesterol and triglycerides suggest shifts in growth rate and diet as turtles recruit from oceanic to neritic foraging habitats [74, 75]. Therefore, the triglycerides in this study that varied with curved carapace length may be an indication of turtles that recently underwent a shift in diet and growth rate. Mitochondrial phospholipids vary with age and growth in fish; however, our correlation results suggest no such relationship in our sea turtles [32]. We did not find sex/age class variability in our turtles despite our principal component analysis showing some clustering (S1 Fig) likely due to sample size or size classification method [44–47]. However, it is important to note that reproduction in reptiles can have dramatic effects on lipid profiles [12, 76, 77]. Some of the individual variability observed in our profiles could be caused by turtles approaching reproductive maturity and onset of migration, although lipid profiles do not indicate initiation of vitellogenesis [16, 17], and our turtles were small (Table 1). Therefore, we suggest that while the species variability in plasma lipids in sea turtles could be partially due to variability in diet, age, or sexual maturation, phylogeny is likely an important indicator of lipid profiles in sea turtles.

Our study identified lipid profile compositional differences between lipid classes that did and did not vary by season. Profiles of structural lipids that displayed seasonality (phosphatidylcholine, phosphatidylinositol, and phosphatidylethanolamine) favored unsaturation compared to saturated lipid species (Fig 2). While phosphatidylcholine profile had a higher proportion of shorter chain lipids, phosphatidylinositol and phosphatidylethanolamine profiles favored longer lipids (Fig 1). These structural lipid classes are related to membrane fluidity [10]. Seasonality in structural lipids suggest that our sea turtles might be displaying homeoviscous adaptation, similar to other amphibians and reptiles [78, 79]. Decreases in membrane rigidity are associated with increases in saturation level of lipids and decreases in lipid chain lengths [9, 13, 78], which maintains trans-lipid transport and metabolism in cold environments. In our study, 22 structural lipids had higher relative ion intensity in the upwelling season when water temperatures were colder (compared to 6 that had higher relative ion intensity in the non-upwelling season; Table 4). Of all lipids, we found more seasonality in hawksbill turtles than in green turtles, suggesting that hawksbill turtles are more affected by upwelling seasonal variability in nutrient availability and temperature than green turtles, potentially due to size differences between populations. Therefore, although sea turtles avoid areas with cold-water [80, 81], seasonal viability in metabolite and lipid profiles may have cold protective abilities that could permit cold-water foraging [56, 82] and minimized migration time [83].

In foraging studies, triacylglycerides, free fatty acids, cholesteryl ester, and acyl-carnitines are the classes used to predict foraging and metabolism [6]. We recorded lower relative ion intensities in triacylglycerides (n = 5), free fatty acids (n = 1) and cholesteryl esters (n = 2) during the non-upwelling season, suggesting slight decreases in foraging when upwelling decreases [6, 84, 85]. In addition, starvation in reptiles is accompanied by a shift from saturated to unsaturated free fatty acids [6, 84], while only one free fatty acid varied by season in this study (and only in hawksbill turtles), it was a highly unsaturated lipid (C22:6; Table 4). However, these conclusions are based on minimal observations (triacylglycerides = 4; free fatty acids = 1; cholesteryl ester = 2) and most of our data suggests consistency in foraging between seasons despite potentially dramatic water temperature changes.

In this study we investigated lipid profiles and relative ion intensities, as opposed to concentrations of specific lipids. This method does not allow us to specifically compare lipid concentrations between our study and the few other studies that have measured lipid concentrations. More specific targeted analysis, such as double bond position in glycerolipids fatty acyl chains [86, 87], is the next step to investigating lipid dynamics in sea turtles. Without specific lipid concentration, we were careful to limit our conclusions to trends in lipid expression and avoid definitive statements about overall amount of lipid present. Further, with the analytical strategy used here, we can only compare lipids within classes and cannot compare lipids across lipid classes. We have identified which lipids are present in sea turtle plasma and using this information, future analysis should build on this work and involve targeted concentrations or variability in genetic expression of specific lipids in sea turtles. Specifically, we suggest investigation of different conditions in sea turtles such as reproduction [12, 16], habitat [2, 79], captivity [61], foraging, body composition and health [31, 46, 88, 89]. Additionally, comparative studies between tissue types may provide insight into gross physiology [90]. Finally, sea turtles are exposed to unique environments as they dive and surface and lipid profiling may help scientists investigate dive capacity in sea turtles [26, 91]. In this study we included ~8 plasma samples with hemolysis, while Stacy et al. [92] suggests hemolysis can alter plasma concentrations of cellular components such as proteins and potassium, they did not quantify the effects of hemolysis on lipid concentrations. Physiologically, it is more likely that it is the lipid presence in blood that causes hemolysis [93]. If we removed samples with hemolysis, our lipid profiles may be biased. Because we processed all samples similarly, the lipid profiling should reflect the physiological state of the turtles. Future research should address whether hemolysis influences lipid profiles in sea turtles.

## Conclusions

To address the limited literature on lipid profiles in the plasma of sea turtles, we report lipid profiling results including chain lengths and saturation levels of a diverse group of lipid classes (sphingomyelin, phosphatidylcholine, free fatty acids, cholesteryl esters, phosphatidylserine, phosphatidylinositol, phosphatidylglycerol, phosphatidylethanolamine, ceramides, and triacylglycerides), and one metabolite group (acyl-carnitines) to support future research into sea turtle lipidomics. We observed a general consistency in the composition of lipid profiles in sea turtles foraging in Pacific Costa Rica, with 20% of lipids measured having significantly different relative ion intensity between either season or species. Our results suggest that lipid profiles are determined partially by phylogeny, but may have a diet, maturation, or habitat component as well. Further, based on foraging lipids analyzed, we suggest that foraging is more consistent in green turtles throughout the year than hawksbills, with lower relative ion intensities present in the non-upwelling season. Finally, we suggest that lipid profiles indicated profile changes between seasons that are consistent with homeoviscous adaptation to changing water temperature. MRM-profiling is a powerful tool allowing researchers to evaluate wildlife foraging across habitats and over time, which has implications for both the future reproductive output of the population and ecosystem management [94, 95]. This study provides the baseline for extending MRM-profiling to sea turtles through a simple, fast, and minimally invasive technique that fosters future investigations of sea turtle health, physiology, and ecology.

## Supporting information

S1 Fig Partial least-squares discriminant analysis displaying age and sex clusters by plasma lipid profiles. Partial least-squares discriminant analysis (PLS-DA) component 1 (x-axis) and component 2 (y-axis) demonstrating age class and sex clusters as a result of plasma lipid profile in green turtles (CM). We live captured all turtles in North Pacific Costa Rica in 2017. Abbreviations: CE = cholesteryl ester; PC = phosphatidylcholine; PE = phosphatidylethanolamine; SM = sphingomyelin; PI = phosphatidylinositol; TG = triacylglyceride; Cer = ceramide; FFA = free fatty acids; PG = phosphatidylglycerol; PS = phosphatidylserine; Car = acyl-carnitine.
(TIF)

S1 Table Tentative identification of lipids and metabolites. Tentative identification of all lipids and metabolites present in sea turtle plasma (relative ion intensity higher than the blank). Lipids and metabolites with significantly different relative ion intensity between species or season are bolded. Abbreviations: CE = cholesteryl ester; PC = phosphatidylcholine; PE = phosphatidylethanolamine; SM = sphingomyelin; PI = phosphatidylinositol; TG = triacylglyceride; Cer = ceramide; FFA = free fatty acids; PG = phosphatidylglycerol; PS = phosphatidylserine; Car = acyl-carnitine.
(XLSX)

S2 Table Average relative ion intensity of seasonal lipids in black morphotype green turtles. Average relative ion intensity of plasma lipids that varied by season (NUP = non-upwelling; UP = upwelling) in black morphotype green turtles, and plasma lipids that varied by morphotype in green turtles (black and yellow). PC = phosphatidylcholine; SM = sphingomyelin; PG = phosphatidylglycerol.
(XLSX)
