# Supplementary figures and images for: Lipid profiling suggests species specificity and minimal seasonal variation in Pacific Green and Hawksbill Turtle plasma
Source: PLoS One. 2021 Jul 19;16(7):e0253916. doi: 10.1371/journal.pone.0253916 (PMC8289036; doi:10.1371/journal.pone.0253916)

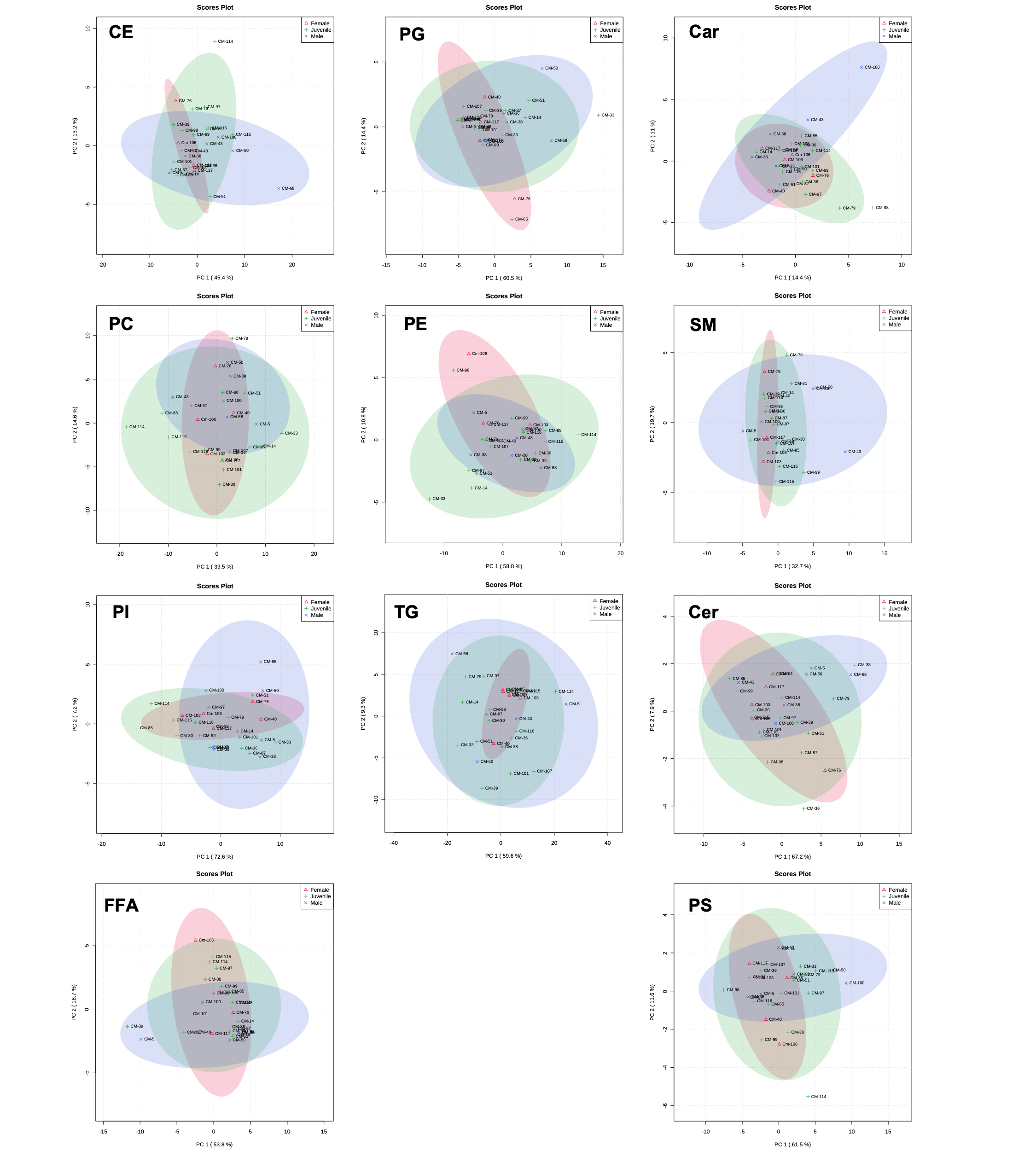

Supplement: S1 Fig — Partial least-squares discriminant analysis (PLS-DA) component 1 (x-axis) and component 2 (y-axis) demonstrating age class and sex clusters as a result of plasma lipid profile in green turtles (CM). We live captured all turtles in North Pacific Costa Rica in 2017. Abbreviations: CE = cholesteryl ester; PC = phosphatidylcholine; PE = phosphatidylethanolamine; SM = sphingomyelin; PI = phosphatidylinositol; TG = triacylglyceride; Cer = ceramide; FFA = free fatty acids; PG = phosphatidylglycerol; PS = phosphatidylserine; Car = acyl-carnitine. (TIF) [file pone.0253916.s001.tif]
